# Clinical Resistance to Lenacapavir During Treatment of HIV Infection: A Review

**DOI:** 10.3390/v18080867

**Published:** 2026-08-08

**Authors:** Nicolas A. Margot, Christian Callebaut

**Affiliations:** Clinical Virology, Gilead Sciences, Inc., 333 Lakeside Dr, Foster City, 94404 CA, USA

**Keywords:** lenacapavir, capsid, HIV-1, resistance testing

## Abstract

Lenacapavir (LEN) is the first-in-class capsid inhibitor that inhibits HIV capsid function in vitro with picomolar activity. LEN has been approved for the treatment of HIV in combination with other antiretroviral agents (ARVs), as well as for the prevention of acquisition of HIV-1 in people who may benefit from pre-exposure prophylaxis (PrEP). In vitro investigations of the resistance profile of LEN have identified resistance-associated mutations (RAMs) at six residues in the HIV-1 capsid protein (CA), all found in the CA structural pocket where LEN binds and conferring LEN-resistance with various degrees of loss of susceptibility. LEN was initially evaluated in a clinical study (CAPELLA) of heavily treatment-experienced (HTE) people with HIV (PWH), in which 14 of 72 participants had emergence of in vitro-predicted LEN RAMs. Despite the presence of LEN RAMs in these participants with viral rebound, treatment with LEN led to viral suppression in a large majority of HTE PWH in CAPELLA. In treatment-naïve participants receiving subcutaneous LEN + asynchronous oral ARVs (CALIBRATE) 4 of 157 participants had emergence of LEN RAM after >2 years of study. In contrast, no cases of viral rebound with resistance to LEN have been observed in virologically suppressed PWH switching to the once-daily single-tablet regimen (STR) combining LEN with the integrase strand-transfer inhibitor (INSTI) bictegravir after up to 48 weeks in two Phase 3 studies, and for >3 years in Phase 2. Similarly, no resistance to LEN has been observed after up to 96 weeks in the Phase 2 study of once-weekly regimen of the deoxyadenosine analog RT inhibitor islatravir + LEN. Finally, in the Phase 2 study of the 6-monthly injectable LEN + 2 broadly neutralizing antibodies (bNAbs) combination, only one instance of resistance to LEN was observed in conjunction with loss of susceptibility to one of the bNAbs through 1 year of treatment. Overall, resistance to LEN in regimens using synchronous dosing (daily or weekly oral, or 6-monthly injectable) has been rare in clinical studies. As the use of LEN is predicted to increase in the near future with these potential new combinations, capabilities to test for capsid resistance are being developed through global commercial options as well as through regional health institutions.

## 1. Introduction

**Figure 1:**
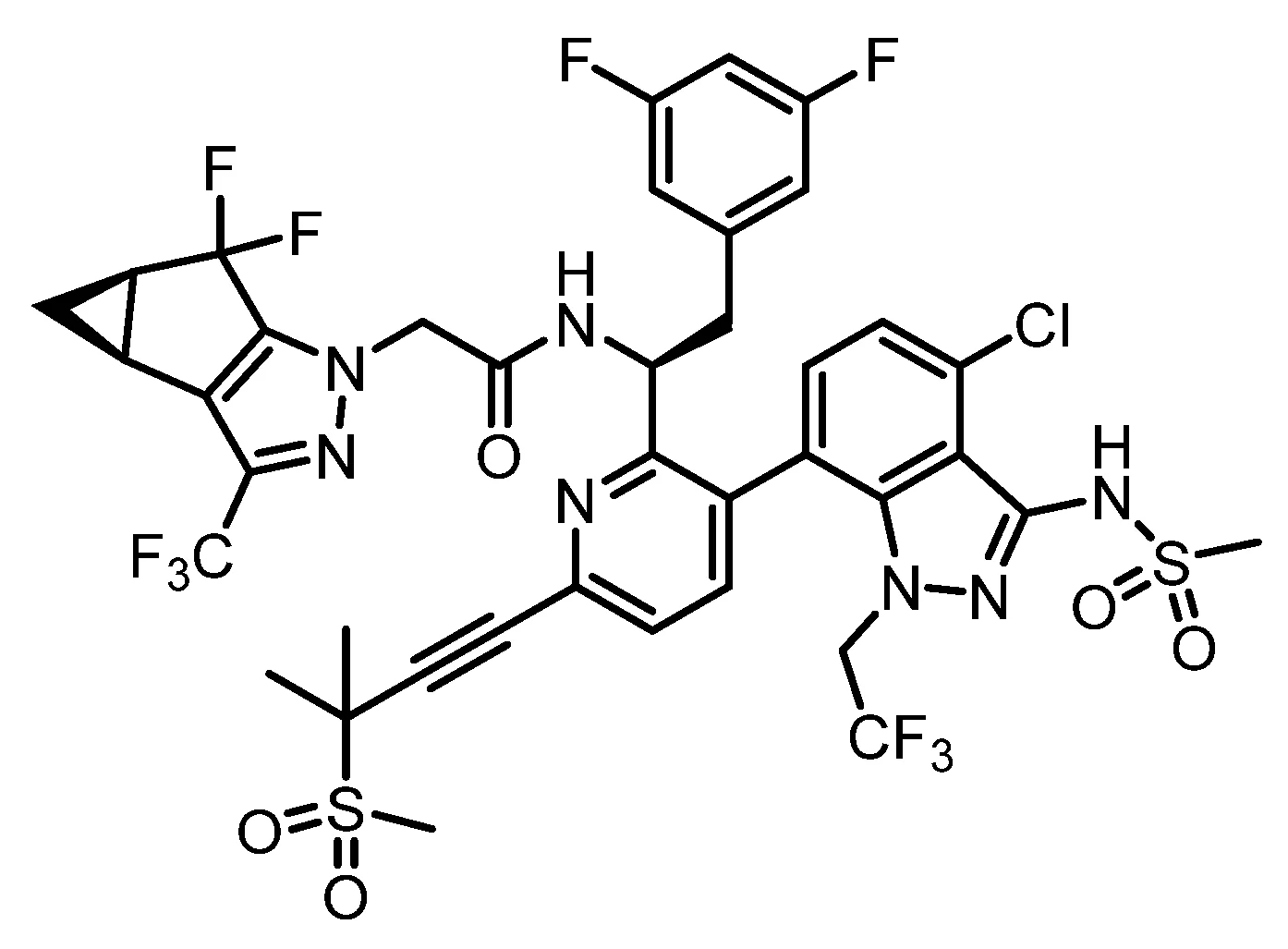
Chemical structure of lenacapavir.

One of the latest medicinal advances in the fight against HIV has been to target its viral capsid. The HIV capsid plays multiple key roles in the viral replication, including protecting the viral RNA after entry into the target cell while reverse transcription is taking place, active transport of HIV genetic material through the cytoplasm via microtubules to the nucleus, facilitating nuclear import and access to integration site, and virus maturation after egress from the infected cell (reviewed in [1,2]). Inhibition of capsid function and overall inhibition of HIV-1 replication has been demonstrated in vitro and clinically with the advancement and approval of lenacapavir (LEN) (Figure 1), as the first-in-class capsid inhibitor (CAI) for the treatment of people with HIV (PWH) with multidrug-resistant (MDR) HIV-1 [3]. Lenacapavir is also approved in several countries for pre-exposure prophylaxis (PrEP) in people who may benefit from PrEP [4,5]. This review provides a brief summary of LEN efficacy and documents the emergence of resistance to LEN in PWH observed to date in clinical studies across the various LEN clinical programs.

## 2. LEN In Vitro Characteristics

Early in vitro studies have demonstrated that LEN inhibits HIV replication with picomolar potency, with an effective concentration to inhibit 50% of viral replication (EC_50_) varying from 32 to 105 pM depending on cell type [3]. This potent antiviral activity is coupled with a lack of cellular toxicity in target cell lines and peripheral blood mononuclear cells (PBMCs) leading to selectivity indices over 1,000,000. Owing to the novelty of the target, LEN has been shown to exhibit wild-type (WT) potency against viruses with high degrees of resistance against all major classes of antiretroviral (ARV) drugs [6,7], demonstrating an orthogonal resistance profile compared to existing ARV drugs. Notably, LEN showed full activity against viruses with resistance to compounds from the maturation inhibitor class, underlining the distinct mechanism of action of LEN compared to maturation inhibitors [7]. In addition, LEN has shown similar picomolar antiviral activity across all HIV-1 subtypes tested [3,8], indicating pan-subtype activity that is partly driven by the high degree of conservation of the capsid protein (CA) across HIV-1 subtypes/clades [9]. LEN also demonstrated antiviral activity against HIV-2 [3], which was further characterized to be reduced 11- to 16-fold compared to HIV-1 [10]. Analyses of LEN pharmacokinetic properties in preclinical studies indicated a long half-life that was dependent on dose, formulation, and species. In rats, doses ranging from 10 to 200 mg/kg led to observed LEN half-lives of 219 to 403 h [11]. These PK properties translated into a half-life of 8 to 12 weeks upon subcutaneous injection of LEN (927 mg) in humans [12].

In vitro resistance to LEN has been studied in dose-escalation and viral breakthrough resistance selection experiments in T cell lines and PBMCs. Seven mutations in CA were selected (starting after 22 days and beyond) in these experiments (L56I, M66I, Q67H, K70N, N74D/S, and T107N) [3], with LEN phenotypic fold changes ranging from 4 to >3200 depending on the mutation tested [13]. The M66I CA mutation was associated with the highest reduction in LEN susceptibility, which was also associated with severely impaired replication of the virus in vitro (<10% of WT). In contrast, the Q67H CA mutation provided near-6-fold reduction in LEN susceptibility with limited impact on the ability of the Q67H virus to replicate [13]. Overall, the level of reduced susceptibility to LEN in the presence of CA mutations was inversely related to the ability of the mutated virus to replicate [3,13,14]. Importantly, LEN resistance-associated mutations (RAMs) are seldom found in PWH at baseline, with a recent baseline prevalence estimate of 0.12% (62 of 52,927) [15].

**Figure 2:**
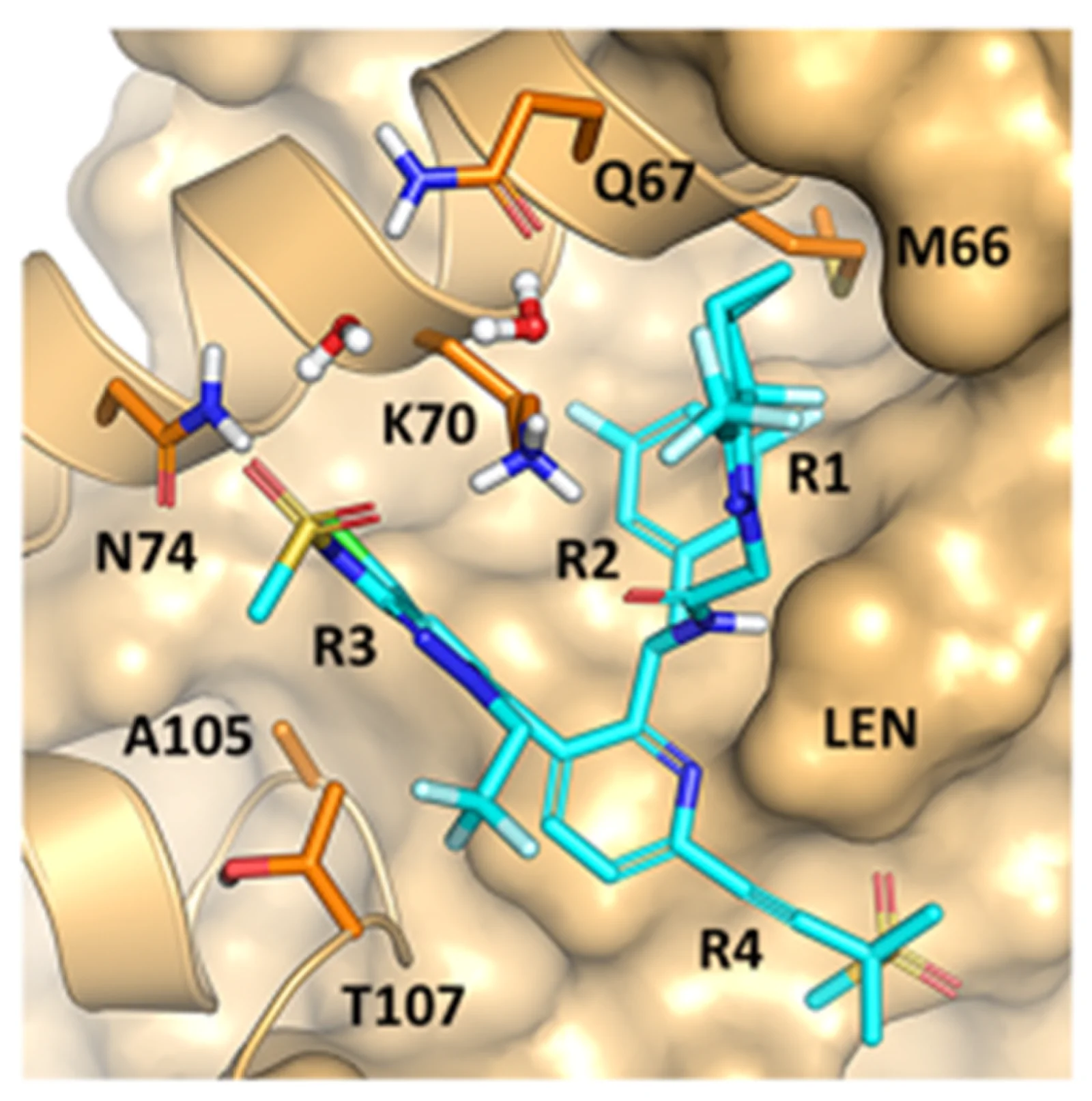
Lenacapavir binding pocket. Lenacapavir, shown in cyan, binds at the interface between two capsid proteins (PDB: 6V2F). Residues associated with clinical resistance to LEN are indicated in orange. Previously published figure [14] reproduced with permission.

The mechanism of action by which LEN inhibits HIV is multifactorial and is thought to operate through stabilization of the capsid lattice at key moments in the HIV life cycle impacting nuclear import and virus maturation [16,17]. Structural studies have revealed that LEN binds into a pocket formed between two adjoining subunits (CA monomers) of the CA hexamer, with six binding sites occupied by LEN [3,17]. The CA mutations selected in vitro all mapped to the LEN binding pocket (Figure 2), providing a strong rationale for the observed mutations and their corresponding levels of LEN reduced susceptibility. Binding studies have indicated that the M66I mutation led to a loss of LEN binding, therefore explaining the high level of reduced susceptibility observed with that mutation [3]. In addition to LEN, cellular cofactors such as cleavage and polyadenylation specificity factor 6 (CPSF6) and nucleoporin 153 (NUP153), which play key roles in nuclear import and integration, also bind to the same CA pocket as LEN, although their binding into the pocket do not appear to be affected by the presence of M66I [18].

## 3. Early Clinical Development of LEN

Physicochemical attributes of LEN (low aqueous solubility, low predicted clearance, and high potency) have enabled the development of LEN as a long-acting agent with infrequent dosing (reviewed in [19]). The phase 1b proof-of-concept study (GS-US-200-4072, NCT03739866) enrolled treatment-naïve (TN) or treatment-experienced (never exposed to CAI or integrase strand-transfer inhibitor [INSTI], and not receiving HIV treatment for >12 weeks before screening) participants who received 1 subcutaneous dose of LEN (20, 50, 150, 450, or 750 mg of LEN suspension formulation) or placebo, and viral load changes were monitored for 10 days. At day 10, all participants were switched to oral once-daily bictegravir/emtricitabine/tenofovir alafenamide (B/F/TAF) for a 225-day follow-up period to provide standard-of-care treatment and prevent long-term exposure to LEN monotherapy for the estimated time period when LEN could exert residual selective pressure. At day 10, viral load declines of 1.4, 1.8, 1.8, 2.2, and 2.3 log [3,13] were observed in the five dose groups, respectively, demonstrating high efficacy of LEN and paving the way for further clinical evaluation. In that study, the Q67H CA LEN RAM was observed in two participants (one in 20 mg and one in 50 mg dose groups), at doses later defined as subtherapeutic [3,13].

## 4. Phase 2 and Phase 2/3 Pivotal Clinical Studies: Approval of LEN

As the first drug in the new class of capsid inhibitors, and owing to its orthogonal resistance profile compared to existing drug classes, clinical studies of LEN were initially focused on PWH with unmet medical needs associated with multidrug-resistant (MDR) HIV-1. The approval of LEN for the treatment of heavily treatment-experienced (HTE) PWH with MDR in combination with other ARVs was supported by the phase 2/3 Study GS-US-200-4625 (CAPELLA, NCT04150068) conducted in HTE PWH with MDR, with Study GS-US-200-4334 (CALIBRATE, NCT04143594) providing additional safety and efficacy data in TN participants. In these studies, participants initially received oral LEN for 2 weeks (600 mg on day 1 and day 2, and 300 mg on day 8), followed on day 15 by a 927 mg subcutaneous dose of LEN that was repeated every 26 weeks, in addition to an optimized background regimen (OBR; Study CAPELLA) [20] or oral ARVs (Study CALIBRATE) [21] starting on day 1. The addition of LEN to an OBR in CAPELLA led to a durable virological response with 85% of participants (44/52, missing = excluded), achieving HIV-1 RNA < 50 copies/mL through 156 weeks of study, along with an increase in CD4+ T-cells of 164 cells/µL [22]. In the TN study, 98–100% (missing = excluded) of participants receiving LEN had HIV-1 RNA <50 copies/mL through Week 132 [23]. Overall, these two studies demonstrated that LEN is highly efficacious in TN PWH as well as in the difficult-to-treat population of HTE PWH with MDR HIV.

Despite the high level of efficacy in CAPELLA, resistance to LEN was detected in 14 of 72 HTE participants with MDR HIV (19%) through 3 years of treatment, with observed CA RAMs M66I (six participants), Q67H/K/N (eight participants), K70H/N/R/S (seven participants), N74D/H/K (three participants), A105S/T (four participants), and T107A/C/N/S (three participants) [24], mostly predicted from in vitro analyses (Table 1). In-depth analysis of the cases of resistance indicated that the 14 participants with LEN RAMs were receiving functional LEN monotherapy at the time of resistance emergence, due either to inadequate adherence to the OBR in 10 participants (OBR drugs not detected in longitudinal plasma samples) or to an OBR containing no active agents in four participants (participant’s HIV resistant to all drugs in OBR) [25]. As a result of the high selective pressure against LEN, the only drug present or active in these participants, LEN RAMs emerged with phenotypic resistance reaching moderate to high levels [14]. Importantly, most LEN RAMs, as single mutations or in combination, were associated with replication defects that were significantly more pronounced than RAMs from other classes, such as the RT RAM M184V [14]. This emphasizes the trade-off for the virus that, becoming resistant to LEN in situation of LEN functional monotherapy, leads to reduced infectivity. Five of the 14 participants with LEN RAMs resuppressed HIV-1 RNA < 50 copies/mL upon OBR switch (two participants) or upon resumption of OBR adherence (three participants) while receiving LEN [24].

In contrast to CAPELLA, LEN RAM emergence in CALIBRATE was much more rare, affecting 4 of 157 TN participants (2.5%) through Week 132 of study, with CA RAMs limited to Q67H alone or the combination Q67H and K70R [23]. Here too, inadequate adherence to the oral components of the regimen may have played a role in the emergence of resistance. One important aspect of treatment with LEN in CAPELLA and CALIBRATE was that the components of the regimens were administered via different schedules and modalities, theoretically increasing the risk of inadequate adherence to treatment and potential emergence of resistance.

## 5. Development of ARV Combinations with LEN

To alleviate asynchronous dosing, new combination treatments with LEN and partner agents where the medications are administered concurrently are in development, therefore mitigating the risk of partial non-adherence to the components of the treatment. These combinations are studied as treatment switch options in virologically suppressed PWH and include once-daily (LEN + integrase strand-transfer inhibitor [INSTI] bictegravir [BIC]) and once-weekly (LEN + RT translocation inhibitor islatravir) oral single tablet regimens (STRs), as well as once-every-6-months injectable drugs (LEN + 2 broadly neutralizing antibodies [bNAbs]). The most advanced of these programs, BIC/LEN, has shown efficacy comparable to control groups through 48 weeks of treatment in two phase 3 studies (ARTISTRY-1, ARTISTRY-2) in virologically suppressed PWH [26,27], with 754 participants receiving the BIC/LEN (75 mg/50 mg) STR and no instances of LEN RAMs in the very few participants experiencing virologic rebound. In addition, no LEN resistance has been observed in the phase 2 portion of ARTISTRY-1 through >96 weeks (median time on BIC + LEN was 167 weeks in the treatment group with the longest exposure to BIC + LEN [N = 47]) [28], with all participants remaining suppressed at the last time point and very rare cases of transient virologic rebound (Table 2). Regulatory review for the BIC/LEN STR indicated for VS treatment switch, particularly for PWH currently receiving complex treatment regimens due to their inability to be on existing STRs (mostly due to prior resistance; ARTISTRY-1 population), is under way. Longer-term data on ARTISTRY-1 and ARTISTRY-2 phase 3 studies will be forthcoming as the studies have been extended to 144 weeks, with many participants from the control groups switching to the BIC/LEN STR.

The next most advanced program is the weekly oral combination of LEN with islatravir (ISL/LEN) that has recently demonstrated non-inferiority to control groups in two phase 3 studies (ISLEND-1, ISLEND-2) at Week 48 [29], after having shown robust efficacy in phase 2 up to 96 weeks of study with no emergence of resistance to LEN in the rare cases of transient virologic rebound [30]. Finally, the 6-month treatment switch option with subcutaneous LEN and infusion of two bNAbs (teropavimab [TAB] and zinlirvimab [ZAB]) in VS participants is also under phase 2 investigation with a phase 3 program under development. There was one instance of emerging resistance to LEN (Q67H, Table 2) along with loss of susceptibility to ZAB out of three participants with virologic rebound in the phase 2 study [31] (N = 53 receiving LEN + bNAbs), with no LEN resistance detected in the other two participants.

## 6. Assays for Resistance Characterization

A simple method to obtain capsid sequence information has been published [32], and research-use-only (RUO) genotypic assays to evaluate the potential emergence of LEN RAMs in CA have been developed by several laboratories, including Monogram Biosciences (South San Francisco, CA, USA) and Institute for Immunology and Genetics (IIG)/Seq-IT (Kaiserslautern, Germany) (Table 3). Both assays employ similar deep-sequencing technologies, with minor differences in the molecular amplification methods. A commercial version of Monogram’s CLIA-validated assay is expected in the second half of 2026. The development of a proprietary assay is also ongoing at another organization (undisclosed for confidentiality), and a number of laboratories across the world (Table 3) have also established CA genotypic assay platforms to make assays available to the medical community. To help with data interpretation, reference institutions have generated algorithms for the analysis of resistance findings based on the published list of capsid mutations affecting LEN with minor differences in the weight attributed to each mutation across the platforms (Table 4). As the use of LEN through the combinations described above is likely to increase globally in the near future, additional options for resistance testing are poised to become available to potentially characterize changes in CA if cases of HIV-1 RNA rebound are observed in PWH receiving LEN.

## 7. Conclusions

In summary, the genetic components of resistance to LEN in vitro and in vivo are highly correlated, with changes in CA at key positions (M66, Q67, K70, N74, A105, and T107) contributing to reduced LEN susceptibility in vivo. In addition, important consequences on viral replication capacity have been noted with most mutations, particularly for M66I mutants, which provided the strongest reduction in LEN susceptibility. Notably, CA resistance observed in the clinical setting was strongly associated with functional monotherapy with LEN, either because the complex optimized background regimen (OBR) prescribed in combination with LEN was ineffective due to prior resistance to components of the OBR or due to inadequate adherence to the OBR from participants in the clinical study (CAPELLA). In people receiving synchronized oral combination treatment of LEN plus a second ARV, no instance of CA resistance emergence has been observed in clinical studies to date, including after treatment for up to 167 weeks with BIC/LEN in the treatment group with the longest exposure to LEN. In the 6-month injectable phase 2 study of LEN + bNAbs, a single instance of CA resistance emergence was accompanied with loss of susceptibility to one of the bNAbs, which may have increased the resistance selective pressure on LEN.

In conclusion, resistance to LEN in the clinic has been mostly associated with the initial treatment of heavily treatment-experienced viremic people with HIV, while no CA resistance has been observed in large studies of synchronized LEN + BIC treatment or phase 2 study of LEN + ISL in virologically suppressed people with HIV.

## Figures and Tables

**Table 1:** Lenacapavir capsid resistance mutations.

Capsid Mutations *	Capsid Residues						
	L56	M66	Q67	K70	N74	A105	T107
In vitro	I	I	H	N	D	-	N
In vivo	-	I	H/K/N	H/N/R/S	D/H/K	T	N **

(*) Resistance-associated mutations observed in vitro and in vivo. (**) Polymorphisms A/C/S have been observed at position T107.

**Table 2:** Treatment-emergent resistance to lenacapavir in clinical studies in people with HIV.

Clinical Trial	Investigational Treatment	Treatment Population	N	Time Point	Participants with Emergent LEN RAMs		LEN RAMs
					n/Total (%) *	n/Tested (%)	
GS-US-200-4072 Phase 1b	SC LEN	TN/TE	39	Day 10	2/29 (NA) †	2/29 (NA) †	Q67H
GS-US-200-4334 Phase 2 CALIBRATE	SC LEN Q6M + oral ART	TN	183	Week 144	4/157 (3%)	4/9 (44%)	Q67H, K70R
GS-US-200-4625 Phase 2/3 CAPELLA	SC LEN Q6M + OBR	HTE MDR	72	Week 156	14/72 (19%)	14/28 (50%)	M66I, Q67H/K/N, K70H/N/R/S, N74D/H/K, A105T, T107A/C/N/S
GS-US-536-5939 Phase 2	Q6M LEN + bNAbs	VS	80	Week 52	1/53 (2%)	1/3 (33%)	Q67H
GS-US-563-6041 Phase 2	QW ISL + LEN	VS	104	Week 96	0/52 (0%)	0/2 (0%)	None
GS-US-621-6289 Phase 2 ARTISTRY-1	QD BIC + LEN	VS	128	Week 96	0/118 (0%)	0/3 (0%)	None
GS-US-621-6289 Phase 3 ARTISTRY-1	QD BIC + LEN	VS	557	Week 48	0/371 (0%)	0/3 (0%)	None
GS-US-621-6290 Phase 3 ARTISTRY-2	QD BIC + LEN	VS	574	Week 48	0/383 (0%)	0/2 (0%)	None

* LEN containing arms only. † NA: not applicable, doses spanned 20–750 mg across five different groups. ART, antiretroviral therapy; HTE, heavily treatment-experienced; LEN, lenacapavir; MDR, multi-drug resistant; OBR, optimized background regimen; Q6M, once every 6 months; QD, once daily; RAM, resistance-associated mutation; SC, subcutaneous; TE, treatment-experienced; TN, treatment-naïve; VS, virologically suppressed.

**Table 3:** Lenacapavir resistance laboratory testing capability *.

	CROs			Local Academic/Hospital Labs		
	Monogram (LabCorp) ^a^	Seq-IT (IIG) ^b^	Other CRO ^c^	Europe ^d^	Brazil	China ^e^
Assay	Yes	Yes	Yes ^f^	Yes	Yes	Yes
Validation	CLIA	IVDR	CLIA ^f^	IVDR/ IvDO	-	Other ^e^
Commercialization/Availability	2026 Non-EU	2025 EU ^g^	2026 ^f^ Non-EU	2026	N/A	N/A

(*) List as of 30 June 2026. ^a^ https://monogrambio.labcorp.com/?utm_source=dl&utm_medium=rd&utm_campaign=monobio, accessed on 24 June 2026. ^b^ https://seq-it.de/, accessed on 24 June 2026 (IIG: Institute of Immunology and Genetics). ^c^ Not disclosed. ^d^ Assays are available in France (https://hivfrenchresistance.org/, accessed on 24 June 2026), Italy (University of Rome Tor Vergata, San Raffaele hospital), and Switzerland (https://www.virology.uzh.ch/de/services/virauftrag.html, accessed on 24 June 2026), and are planned in Belgium (KU Leuven) and Greece (University of Athens) (personal communication). ^e^ Peking University; data on file. ^f^ Ongoing/planned. ^g^ Currently limited to Germany. CLIA, Clinical Laboratory Improvement Amendments; CRO, contract research organization; EU, Europe; IvDO, in vitro medical devices diagnostic ordinance; IVDR, in vitro diagnostic regulation; N/A, not applicable; RUO, research use only.

**Table 4:** HIV-resistance algorithms.

Reference Institution	Status	Accessibility
Stanford University (USA)	Public	https://hivdb.stanford.edu/ (last accessed on 24 June 2026)
GRADE (Germany)	Public	https://www.hiv-grade.de (last accessed on 24 June 2026)
ANRS (France)	Public	https://hivfrenchresistance.org (last accessed on 24 June 2026)
IAS-USA (USA)	Public	Yearly publication [33]
Monogram Biosciences (USA)	Proprietary	N/A

N/A: not applicable.

## Data Availability

No new data were created or analyzed in this study. Data sharing is not applicable to this article.
